# ESMPE: A combined strategy for school tuberculosis prevention and control proposed by Dalian, China

**DOI:** 10.1371/journal.pone.0185646

**Published:** 2017-10-03

**Authors:** Xichen Wang, Hongbo Jiang, Xuemei Wang, Hongyu Liu, Ling Zhou, Xiwei Lu

**Affiliations:** 1 School of Public Health, Dalian Medical University, Dalian, Liaoning, China; 2 Office of Epidemic Surveillance, Dalian Tuberculosis Hospital, Dalian, Liaoning, China; Indian Institute of Technology Delhi, INDIA

## Abstract

**Background:**

Although China has paid more attention on the prevention and control of tuberculosis (TB) in schools, several unsolved questions in this field still threaten the progress of TB control. Therefore, there is an urgent need to develop a systematic and practical strategy for Chinese school TB prevention and control system. In this study, we aimed to assess the feasibility of a combined strategy named ESMPE (examination, screening, monitoring, prevention and education) that adhere to the basic principles of Chinese schools TB control strategy.

**Methods:**

The ESMPE strategy included five sections, namely TB screening during physical examination for the school freshmen entrances, screening of close contacts, monitoring of high-risk schools, preventive treatment and TB education. The effectiveness of ESMPE strategy was evaluated from 2011 to 2016. The original data were provided by the Dalian Tuberculosis Hospital. Descriptive analysis and nonparametric tests were used for comparing statistical differences of results between different years.

**Results:**

The detection rate of active pulmonary TB in school freshmen was decreased from 2011 to 2016 (*χ*^*2*^ = 41.941, *P* = 6.0551E-8). 97.22% (17,043/17,530) of close contacts experienced close contacts screening, and the secondary attack rate (SAR) of TB in schools fell by 146.35/10^5^ from 2011 to 2012, and finally reduced to 85.57/10^5^ in 2016. There was a significant correlation between SAR of student TB and the rate of screened close contacts (*r* = -0.924, *P* = 0.009). TB incidence of five monitored schools had a substantial decline after receiving monitoring, and this declining trend continued in 2016. Due to the TB education and advanced screening methods, the mean of diagnostic delay time in students with TB was shortened (15.71 days), while still fewer latent TB infection students received preventive treatment (30.38%).

**Conclusions:**

The ESMPE strategy has shown a favorable effect on TB prevention and control in Dalian schools. More systematic evidence is needed on the effect of this strategy in reducing the incidence of TB in schools from other settings prior to its further scaling-up in China.

## Introduction

China is a country with high burden of tuberculosis (TB), with about 1.06 million new cases annually, accounting for 10% of the global incidence and ranking third worldwide [1]. The fifth national TB survey conducted in 2010 showed that the prevalence of active TB was 459/100,000 when compared with the prevalence of 466/100,000 in 2000, however, it is estimated that 44.5% of China’s population were infected with TB [2], which represents an enormous challenge to the public health. Many researchers pointed out that students were at high risk for TB infection [3]. According to the data from National Internet-based Infectious Disease Reporting System in 2014, 4.02% of TB patients were students and the incidence of school TB was 20.16/10^5^ (41,608/206,339,584) in China [4]. In addition, students had been confirmed as a high-risk group for clustered TB, the incidence of which often substantially exceeded the average level of the local society once outbreaks have started [5].

The Chinese government has paid more attention on the prevention and control of TB among student population [6]. In 2012, the *national guidelines for prevention of school tuberculosis* was jointly formulated by the Ministry of Health and Ministry of Education, which clarified the responsibility of school in TB control strategy. TB screening for the school freshmen entrances and TB close contacts were recommended as effective measures to identify TB cases in student population at early stage. In addition, the student with no indication for chest radiography while positive tuberculin skin test (TST) result with a duration of ≥15 mm was deemed as latent tuberculosis infection (LTBI). However, the frequent emergence of school TB outbreaks challenges the current TB control strategies for student population [7]. On one hand, the strategies regarding the management of TB outbreak in school are still lacking. On the other hand, the imbalanced development of economic across China leads to the inefficient implementation of the strategies in the low economic cities. Therefore, the unsatisfactory situation highlights the urgent need to develop a more universal and effective strategy to impede the TB epidemic in schools.

Dalian is an relatively developed city in the northeast of China. The city has more than 900 schools and 930,000 students, and the proportion of TB epidemic in schools accounted for almost 6.55% of TB incidence of this city. In view of the high TB burden in this population, Dalian established a distinctive ESMPE (examination, screening, monitoring, prevention and education) strategy for school TB prevention and control based on six years of exploration and practice. In this study, we retrospectively analyzed the data of school TB incidence in Dalian from 2011 to 2016. Our aim was to assess the feasibility of this innovative strategy to reduce the TB incidence in schools in Dalian.

## Materials and methods

### Study design

The effectiveness of the ESMPE strategy was evaluated by analyzing the implementation data. This strategy includes five following aspects: TB screening during physical examination for the school freshmen entrances; screening of close contacts; monitoring of high-risk schools; preventive treatment; TB knowledge education. The concept of the ESMPE strategy is completely proposed by the research team independently.

**Fig 1:**
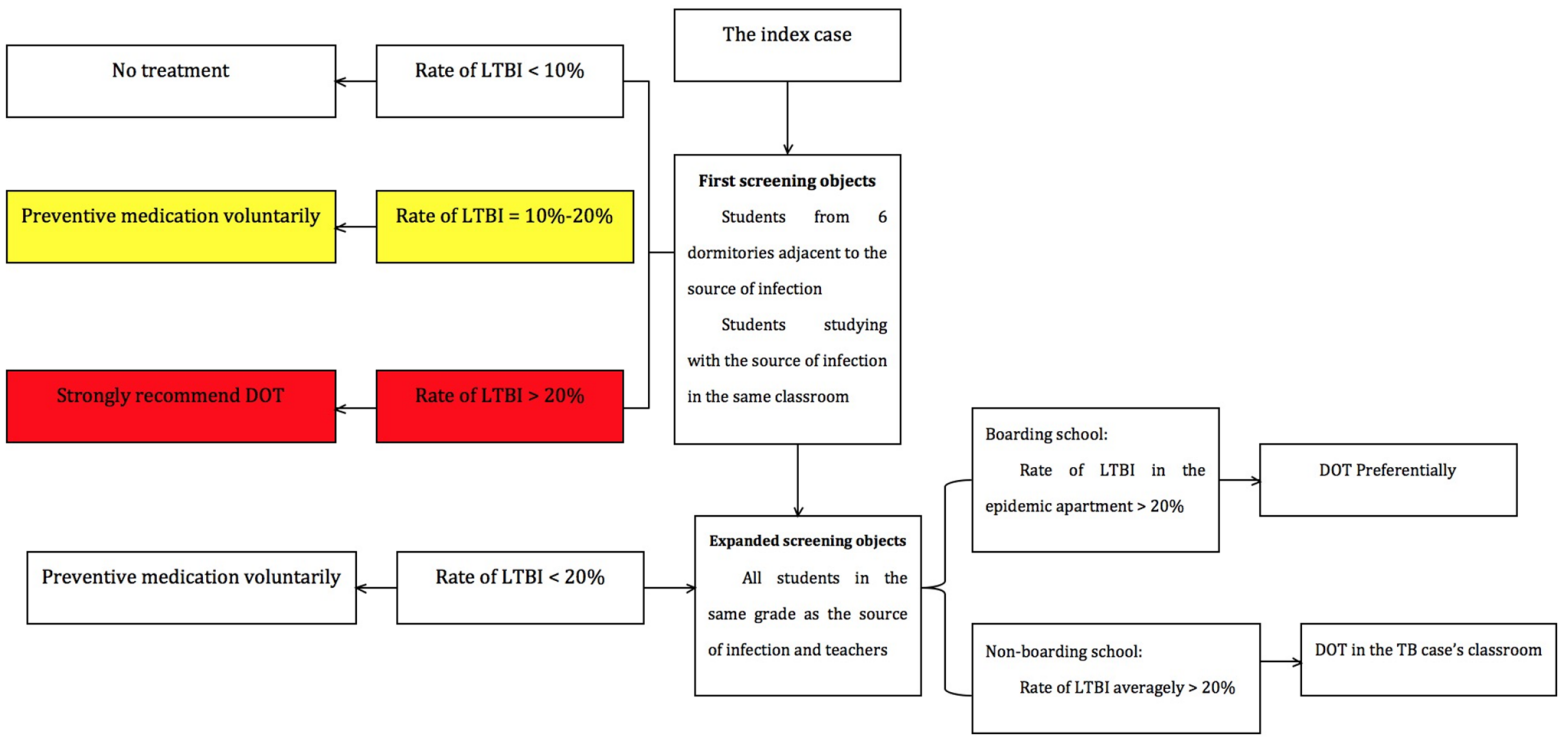
Screening and management procedures of TB close contacts.

Firstly, TB screening during physical examination for the school freshmen entrances in Dalian mainly includes two parts: one part was to find out LTBI in school among freshmen by TST, and these LTBIs determined by TST were recommended to have chest radiography after six months; and the other part was to diagnose active pulmonary TB cases using chest radiography, and then these patients received anti-TB treatment immediately. The examination was usually performed between September and October per year, which was incorporated with physical examination during registration of freshman students. Secondly, TST and chest radiography were also used for screening the close contacts. A standardized screening and management procedures of TB close contacts had been established since 2012 in Dalian (Fig 1). Briefly, the close contacts were divided into three level based on potential exposure to active TB, including high, medium and low level [8]. The students living in the same or neighboring classroom and dormitory were at high exposure level; the students living in the same floor but not belonging to high exposure level group were at medium exposure level; the other students were at low exposure level. The students experienced high or medium exposure level were screened due to cost-effectiveness considerations. Thirdly, monitoring of high-risk schools focused on the students living in the schools with high TB incidence. Compared with the routine screening among freshman students, all the people from schools with the top five TB incidence last year had chest radiography at the beginning of this year. If an active TB was diagnosed by chest radiography, students from this school would experience TB physical examination. Fourthly, preventive treatment for LTBI was an important strategy for preventing the subsequent development of active TB. The treatment regimen for LTBI included isoniazid 0.3 g daily plus rifapentine 0.6 g twice weekly [9]. For the patients with body weight less than 50 kg, the dosage of rifapentine was adjusted to 0.45 g twice weekly [9]. Fifthly, we carried out active TB education among the student population in Dalian. The content of TB education included the knowledge of TB diagnosis and treatment, as well as the national strategy for TB control and prevention. The five strategies mentioned above constituted the ESMPE strategy.

### Data collection

Dalian Tuberculosis Hospital was the public health sector mainly responsible for TB control and prevention in Dalian. Data on reported TB cases from the schools of Dalian were obtained from national TB reporting system between 2011 and 2016. In addition, numbers of registered freshman students, index cases, and close contacts from schools in Dalian were collected for further analysis, respectively. The intervals between initial symptoms and final diagnosis as active cases were also recorded. Based on these data, several indicators were proposed to evaluate the effectiveness of ESMPE strategy, including the secondary attack rate (SAR) and interval of diagnostic delay.

### Data analysis

Data were entered by using EPIDATA 3.1 and analyzed by using MS Excel (Microsoft Corporation, Redmond, State of Washington) and SPSS 22.0 statistical package (IBM Corporation, Armonk, State of New York). Pearson correlation analysis was used for the normal distribution data, and nonparametric tests (Chi-square test and Kruskal-Wallis test) were used for non-normal distribution data. Statistical significance was declared as significant if *P* value was less than 0.05.

## Results

Table 1 shows the results of TB screening during physical examination for the school freshmen entrances in Dalian from 2011 to 2016. A total of 999,209 school freshmen had the TB screening during physical examination within six years, accounting for 75.13% of the freshmen. The proportion of freshmen receiving TB screening was increasing except for the data from 2013, which was mainly due to the lack of TST in China. The strongly positive rate of TST and detection rate of active pulmonary TB in school freshmen were significantly decreased from 2011 to 2016 (*χ*^*2*^ = 2273.782, *P* = 3.0938E-139 for strongly positive rate of TST; *χ*^*2*^ = 41.941, *P* = 6.0551E-8 for detection rate of active pulmonary TB, respectively).

**Table 1:** Results of TB screening during physical examination for the school freshmen entrances from 2011 to 2016.

Year	Enrollment of freshmen	Accepted TB screening		TST≥15mm		Active pulmonary TB	
		n	%	n	%	n	1/10^5^
2011	211035	153493	72.73	7666	4.99	52	33.88
2012	210834	145548	69.03	3434	2.36	57	39.16
2013	216227	127299	58.87	4256	3.34	73	57.34
2014	229500	179153	78.06	7101	3.96	48	26.79
2015	217650	173789	79.85	5669	3.26	72	41.43
2016	244795	219927	89.84	5746	2.61	41	18.64
Total	1330041	999209	75.13	33872	3.39	343	34.32

We further analyzed the data regarding index cases, close contacts and secondary cases in Dalian schools from 2011 to 2016 (Table 2). The proportions of screened cases and screened close contacts were increased since the screening and management procedures was implemented in 2012. The SAR of TB in schools fell by 146.35/10^5^ from 2011 to 2012, and further reduced to 85.57/10^5^ in 2016. Statistical analysis revealed that there was a significant difference in SAR between 2011 to 2016 (*χ*^*2*^ = 56.813, *P* = 5.5268E-11). In addition, a significant negative correlation between SAR of student TB and the rate of screened close contacts (*r* = -0.924, *P* = 0.009) was observed in this study. We also compared the effectiveness of detection rate of active TB between school freshmen entrance TB screening during physical examination and close contacts screening. The average value of active TB detection rate of close contacts screening in six years was 218.24/10^5^, but of school freshmen entrance TB screening during physical examination was only 36.21/10^5^, with statistical significance (*χ*^*2*^ = 624.354, *P* = 8.4477E-138), which suggests that close contact screening is a more effective way than the others when screening active TB.

**Table 2:** Screening of close contacts and secondary attack rate (SAR) from 2011 to 2016.

Year	Index cases	Screened cases		Close contacts	Screened close contacts		Secondary cases	
		n	%		n	%	n	SAR (1/10^5^)
2011	275	125	45.45	18214	7886	43.30	77	422.75
2012	269	251	93.31	15557	13535	87.00	43	276.40
2013	268	261	97.39	16837	15672	93.08	30	178.18
2014	221	218	98.64	24366	22669	96.38	43	176.48
2015	233	230	98.71	15878	15403	97.01	27	170.05
2016	239	237	99.16	17530	17043	97.22	15	85.57
Total	1505	1322	87.84	108382	92208	85.07	235	216.83

**Fig 2:**
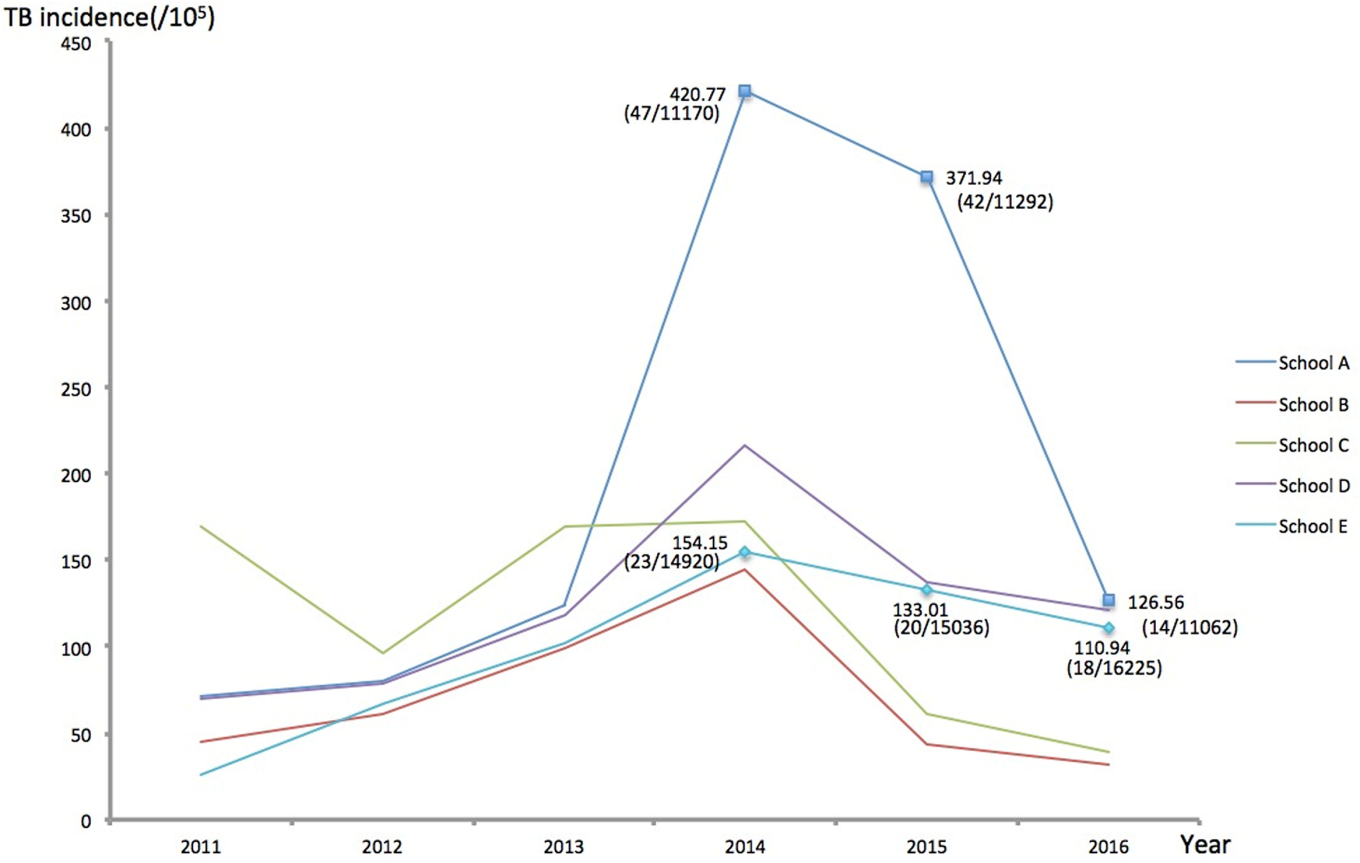
Trend of TB incidence of five high-risk schools from 2011 to 2016.

As shown in Fig 2, the overall TB incidence of five schools had decreased from 209.26/10^5^ (147/70,249) in 2014 to 132.18/10^5^ (99/74,899) in 2015 after the intervention in the schools at high-risk (S1 Table). The greatest decrease in TB incidence among five schools was observed in School A, with a decreased incidence of 294.21/10^5^, whereas the least decrease was identified in School E, with a decreased incidence of 43.21/10^5^. This overall declining trend in TB incidence indicated the potential effectiveness of high-risk schools monitoring.

Table 3 shows the analyzed results of students receiving preventive treatment from 2013 to 2016. A total of 1891 LTBI students (30.38%, 1891/6225) agreed to accept preventive treatment. The proportion of LTBI students receiving preventive treatment varied between 15.75% (244/1549) and 42.92% (582/1356) from 2013 to 2016. Out of 1891 LTBI students receiving preventive treatment, 753 students (39.82%) completed a course of LTBI treatment, whereas the other 1138 students (60.18%) interrupted preventive treatment because of various reasons. In addition, the percentages of LTBI completing preventive treatment from both sporadic and clustered cases were not significantly increased during the past four years. However, this percentage of clustered cases was significantly higher than that of sporadic cases, and the difference was statistically significant (*χ*^*2*^ = 448.892, *P* = 2.4824E-108).

**Table 3:** Analysis of preventive treatment to LTBI students from 2013 to 2016.

Year	LTBI students from close contacts screening	LTBI students receiving preventive treatment	LTBI students from sporadic cases		LTBI studentsfrom clustered cases	
			Receiving treatment	Completing treatment (%) ^a^	Receiving treatment	Completing treatment (%)
2013	1356	582	165	22 (13.33)	417	281 (67.39)
2014	1925	496	155	19 (12.26)	341	166 (48.68)
2015	1395	569	384	52 (13.54)	185	146 (78.92)
2016	1549	244	190	28 (14.73)	54	39 (72.22)
Total	6225	1891	894	121 (13.53)	997	632 (63.39)

^a^ The rate of LTBI students completing preventive treatment among LTBI students receiving preventive treatment.

**Fig 3:**
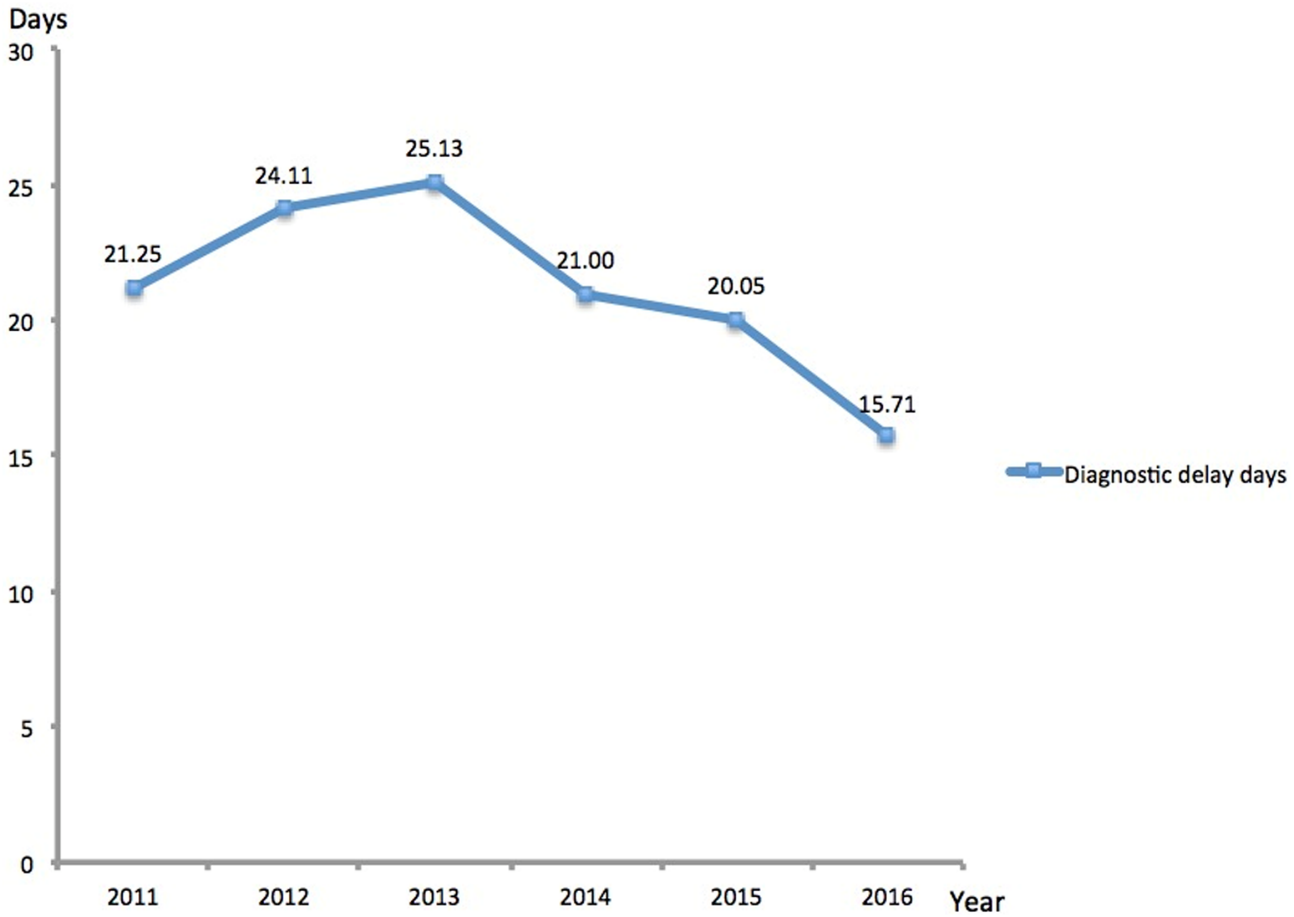
Trend of diagnostic delay days of the diagnosed TB patients from 2011 to 2016.

The intervals of diagnostic delay were used as an indicator to assess the effectiveness of TB education to students [10]. As shown in Fig 3, the intervals of diagnostic delay from 2011 to 2016 were 21.25±40.01 days, 24.11±46.18 days, 25.13±59.27 days, 21.00±32.60 days, 20.05±32.56 days and 15.71±22.78 days, respectively. The difference in the intervals of diagnostic delay had statistical significance (*H* = 24.33, *P* = 0.000187).

**Fig 4:**
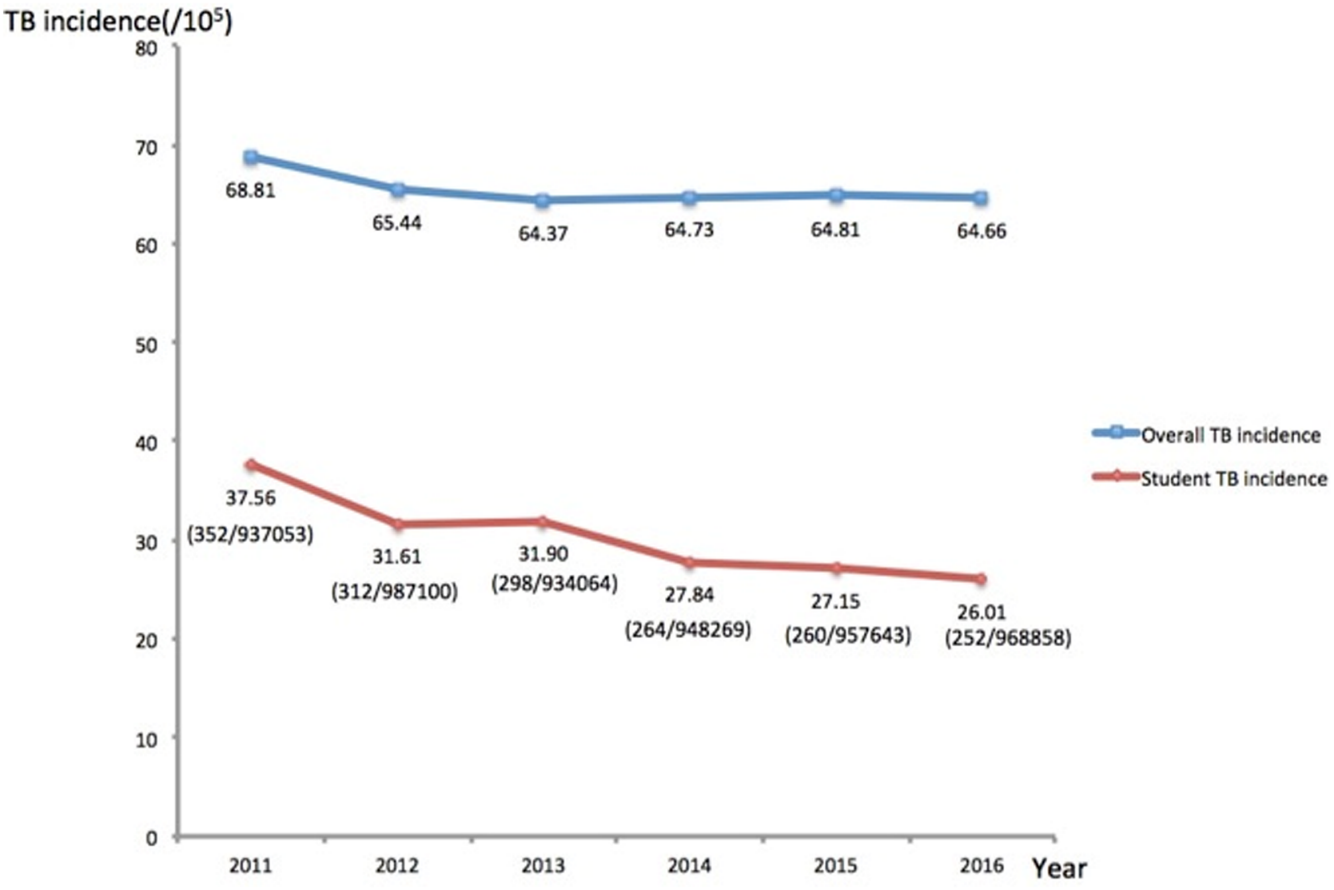
The overall incidence of TB and the incidence of student TB in Dalian from 2011 to 2016.

We also compared the TB incidence between the general population and student population in Dalian from 2011 to 2016 in Fig 4 (S2 Table). The result of Chi-square test showed there was no significant difference in TB incidence during the past six years (*χ*^*2*^ = 0.237, *P* = 0.994). Despite showing a stable trend in general population, statistical analysis revealed that the school TB incidence was significantly decreased from 2011 to 2016 (*χ*^*2*^ = 28.806, *P* = 0.000025). Obviously, this decrease trend could be an indicator for the positive effect of implementing the ESMPE strategy on reducing the TB incidence in schools.

## Discussion

In this study, we firstly established a novel and comprehensive strategy (ESMPE) for TB control and prevention in student population, who are at high risk for TB infection in China. Our primary data revealed that both the rate of active TB and the number of LTBI were decreasing in school population from 2011 to 2016, which is majorly attributed to the effectiveness of ESMPE. In our opinion, this new strategy has several advantages. First, active case finding is the essence of ESMPE strategy. By integrated with physical examination, ESMPE provides the possibility of conducting TB examination among a large number of freshman students. On one hand, all the active cases were identified before entry into school, thus preventing further transmission in the student community. On the other hand, the implementation of close contact screening would find students with TB early enough to reduce the emergence of bacteria positive patient. Notably, the strategy for close contact screening was conducted according to risk stratification of TB infection in the student population, which is a useful model not only to screen close contacts at high and moderate risk but also to produce the favorable cost-effectiveness results. However, in view of the long incubation period of TB, a major effort to conduct follow-up screening for close contacts is needed [11].

Second, in addition to the traditional procedures of close contact screening, we paid more attention to the schools with the top five TB incidence. The screening targets were expanded to all people rather than only freshman students. In order to detect the active cases early enough, we used chest radiography to screen the target people. Of note, on the basis of analyzing the trend of TB incidence in five monitored schools from 2011 to 2016, we found that TB incidence of five schools in 2015 has been a substantial decline after receiving monitoring and intervention activities, indicating the satisfactory effect produced by the monitoring and intervention among the students living in settings with high TB incidence. This measure could be considered as the subsequent screening of close contacts at high risk, and its obvious results in reducing the TB incidence of schools echo the suggestion mentioned above that it is necessary to prevent TB outbreaks through the follow-up monitoring of close contacts prior to the occurrence of cases.

Many studies have demonstrated that preventive treatment is an effective strategy to prevent LTBI from becoming active pulmonary TB patient [12]. The treatment of LTBI is strongly recommended as an effective method for preventing TB in people who show positive TST and who are at risk for reactivated TB [13]. Unfortunately, due to asymptomatic manifestation of LTBI and adverse events associate with the use of anti-TB drugs, the completion rate of preventive treatment among LTBI ranges generally from 43% to 90% [14]. Hence, the improvement of adherence to chemotherapy for LTBI plays an important role in promoting the efficacy of preventive treatment. In line with previous findings, the proportion of LTBI student completing preventive treatment was not satisfactory [15], and only 12% of LTBI students completed preventive treatment in our study. In China, there is no official definition for LTBI to date, and the corresponding guidelines have not been established to conduct preventive treatment for LTBI. In the present study, preventive treatment was supervised by student volunteers rather than physical clinicians. Hence, the relative low rate of LTBI students who had completed preventive treatment probably reflects the unsatisfactory efficacy in witnessing the prescription of anti-TB drugs from poorly trained volunteer. In addition, fear of the adverse event due to the medication of preventive treatment is another important reason for the interruption of preventive treatment [16]. In view of the great threat posed by school cluster epidemic, more attention should be paid on the manage the LTBI students to reduce the potential emergence of new cases and further outbreak in this population. Notably, we found that the percentage of LTBI completing preventive treatment among clustered cases was significantly higher than that among sporadic cases. Given that clustered cases were always associated with outbreak of TB, our primary data may reflect the core role of government support in improving medication adherence in LTBI students. In addition, our previous study have demonstrated that the application of directly observed treatment for student population produced a favorable increase in adherence of preventive treatment, and the rate of students completing treatment increased from 45.6% to 85.9% [17]. Taken together, the management of LTBI students requires the innovative and effective collaboration between health and education department in the future.

TB education is another advantage of this strategy for school students. Providing basic information about the earliest symptoms of TB can shorten the diagnostic delay, and thus strengthen the effect on infection risk of control programs with low TB transmission [18–19]. In this study, the diagnostic delay intervals exhibited significant decrease from 2011 to 2016. Although the application of new diagnostic tools and advanced screening methods are contributors to this declining trend, we speculate that TB education could serve as an important factor associated with the shorter patient delays. In line with our hypothesis, TB education can improve the students’ ability to diagnosis early symptoms of TB by themselves, indicating that students with suspected symptom sought health care in early stage of TB course. Hence, the shortened interval of diagnostic delay is not only related to the favorable clinical outcome for TB individual, but also impedes the transmission of TB in the community. In view of this fact, further strengthening TB education to students will produce additional benefit in promoting the implementation of TB prevention and control strategy for school.

There were several obvious limitations in this study. First, in the ESMPE strategy, not all sections of this strategy started meanwhile, such as preventive treatment in 2013 and monitoring of high-risk schools in 2015. The diverse starting time of different sections may confuse the interpretation of data. Second, in view of high prevalence of TB infection in China, the criteria for LTBI used in this study was the patients with TST≥15 mm, which is different from the international standard of TST≥10 mm. The strict standard may result in loss of detection of LTBI. Third, the students from the kindergartens were excluded from this study due to the enormous difficulty of obtaining a consent form from a parent or legal guardian. Fourth, this study was only conducted in Dalian, a relatively developed prefecture, thus further evaluation is required to be completed in different settings of China prior to the scaling-up of this novel strategy.

## Conclusion

In summary, ESMPE provides a comprehensive strategy for the control and prevention of TB in Chinese schools by combining active TB screening, preventive treatment and TB education. Our primary data have demonstrated that the implementation of this novel strategy promote the reduction in the TB incidence of school in Dalian. Further evaluation is required to be completed in different settings of China prior to the scaling-up of this novel strategy.

## Supporting information

S1 Table Initial data of five high-risk schools from 2011 to 2016. (PDF)

S2 Table Initial data of TB incidence in Dalian from 2011 to 2016. (PDF)
