# Supplementary material for: ESMPE: A combined strategy for school tuberculosis prevention and control proposed by Dalian, China
Source: PLoS One. 2017 Oct 3;12(10):e0185646. doi: 10.1371/journal.pone.0185646 (PMC5626428; doi:10.1371/journal.pone.0185646)
Supplement: S1 Table — (PDF) [file pone.0185646.s001.pdf]

Fig.2 Trend of TB incidence of five high-risk schools from 2011 to 2016 (Initial Data)			
**School**	**Year**	**Number of Students in School**	**Number of TB Students**
School A	2011	11328	8
	2012	11308	9
	2013	12111	15
	2014	11170	47
	2015	11292	42
	2016	11062	14
School B	2011	17925	8
	2012	19617	12
	2013	20133	20
	2014	16664	24
	2015	18302	8
	2016	19017	6
School C	2011	17118	29
	2012	16507	16
	2013	17087	29
	2014	14564	25
	2015	16423	10
	2016	18121	7
School D	2011	13000	9
	2012	14000	11
	2013	14408	17
	2014	12931	28
	2015	13846	19
	2016	14000	17
School E	2011	11834	3
	2012	11841	8
	2013	11797	12
	2014	14920	23
	2015	15036	20
	2016	16225	18
