# Supplementary material for: ESMPE: A combined strategy for school tuberculosis prevention and control proposed by Dalian, China
Source: PLoS One. 2017 Oct 3;12(10):e0185646. doi: 10.1371/journal.pone.0185646 (PMC5626428; doi:10.1371/journal.pone.0185646)
Supplement: S2 Table — (PDF) [file pone.0185646.s002.pdf]

Fig.4 The overall incidence of TB and the incidence of student TB in Dalian from 2011 to 2016 (Initial Data)				
**Year**	**Population (×100000)**	**TB Patients**	**Number of Students**	**Number of TB students**
2011	588. 5	4049	937053	352
2012	590. 3	3863	987100	312
2013	591. 4	3807	934064	298
2014	594. 3	3847	948269	264
2015	593. 6	3847	957643	260
2016	595. 1	3848	968858	252
